# Characteristics and mortality of 561,379 hospitalized COVID-19 patients in Germany until December 2021 based on real-life data

**DOI:** 10.1038/s41598-022-15287-3

**Published:** 2022-07-01

**Authors:** Jan Andreas Kloka, Lea Valeska Blum, Oliver Old, Kai Zacharowski, Benjamin Friedrichson

**Affiliations:** Department of Anaesthesiology, Intensive Care Medicine and Pain Therapy, University Hospital Frankfurt, Goethe University, Theodor-Stern Kai 7, 60590 Frankfurt, Germany

**Keywords:** Medical research, Epidemiology

## Abstract

The ongoing SARS-CoV-2 pandemic is characterized by poor outcome and a high mortality especially in the older patient cohort. Up to this point there is a lack of data characterising COVID-19 patients in Germany admitted to intensive care (ICU) vs. non-ICU patients. German Reimbursement inpatient data covering the period in Germany from January 1st, 2020 to December 31th, 2021 were analyzed. 561,379 patients were hospitalized with COVID-19. 24.54% (n = 137,750) were admitted to ICU. Overall hospital mortality was 16.69% (n = 93,668) and 33.36% (n = 45,947) in the ICU group. 28.66% (n = 160,881) of all patients suffer from Cardiac arrhythmia and 17.98% (n = 100,926) developed renal failure. Obesity showed an odds-ratio ranging from 0.83 (0.79–0.87) for WHO grade I to 1.13 (1.08–1.19) for grade III. Mortality-rates peaked in April 2020 and January 2021 being 21.23% (n = 4539) and 22.99% (n = 15,724). A third peak was observed November and December 2021 (16.82%, n = 7173 and 16.54%, n = 9416). Hospitalized COVID-19 patient mortality in Germany is lower than previously shown in other studies. 24.54% of all patients had to be treated in the ICU with a mortality rate of 33.36%. Congestive heart failure was associated with a higher risk of death whereas low grade obesity might have a protective effect on patient survival. High admission numbers are accompanied by a higher mortality rate.

## Introduction

The ongoing COVID-19 pandemic is affecting people worldwide since the first reported case. Up to the end of October, more than 246 million cases and up to 5 million deaths have been reported^1,2^. The number of unreported cases is probably much higher. This makes the COVID-19 pandemic one of the deadliest in history.

Infection with the SARS-CoV-2 virus presents with a wide variety of symptoms—From none to life threatening. This complicates the detection and containment of the virus. With the worldwide spread of the SARS-CoV-2 virus, a burden has been placed on the health care systems worldwide. Especially the intensive care units (ICU) as a limited resource were occupied with the care of COVID-19 patients. In some countries, the ICU capacities reached their limits^3,4^, resulting in catastrophic outcomes for the patients. Over the pandemic, admission rates to ICU and mortality rate varied strongly. In the early phase of the pandemic, first reports and characterisations based on smaller populations mainly in China suggested only mild symptoms in 80% of the cases^5–8^. 20% needed hospital treatment and 20% of the hospitalized patients required ICU treatment^6,7,9^. Those numbers vary within the European Union^10^. Estimates suggest that between 10 and 20% of SARS-CoV-2 patients become so severely ill, that hospital treatment is required. In addition, the proportion of infected people who require intensive care also varies between 5 and 32%^10,11^.

Therefore, it is important to be able to adequately calculate the resources of the health care system to avoid a collapse of the system in the event of another wave. The present study therefore provides up-to-date data based on which calculations could be made. The aim of this observational study is to describe the dynamic of the pandemic in Germany and identify the underlying characteristics of hospitalized patients from January 2020 until the end of December 2021, based on data from the German Institute for Hospital Remuneration System (InEK).

## Materials and methods

### Inclusion criteria

All hospitalized patients in Germany with proven SARS-CoV-2 infection between January 1st, 2020 and December 31th, 2021 were included.

### Definitions and data acquisition

We divided patients into subgroups in dependence of admission to the ICU or to the general ward (non-ICU) and distinguished in both subgroups between survivors and non-survivors. The collected data included age, comorbidities (congestive heart failure, arterial hypertension, chronic pulmonary disease, diabetes and obesity), complications (acute renal failure, dialysis, cardiac arrhythmias, cardiopulmonary resuscitation (CPR), embolism, thrombosis, myocardial infarction, pulmonary embolism, intracranial hemorrhage and stroke), length of hospital stay and mortality. Due to our findings associated with obesity, we used and subdivided obesity according to the WHO-definition into grade I, II and III. Diagnoses were coded according to the tenth revision of the International Classification of Diseases (ICD) and procedures were coded according to the International Classification of Procedures in Medicine in the version of 2020 (Table 1). The InEK only allows anonymized queries; there is no possibility to track a case back to a patient and therefore no further analysis is possible at the individual case level. In this study, we included only patients with a confirmed SARS-CoV-2 infection by Real-Time-(RT)-PCR (ICD U07.1) and admitted to hospital between January 1st, 2020 and December 31th, 2021.

**Table 1:** DRG and OPS codes.

Comorbitiy	OPS	ICD-10
Arterial hypertension		I10.x, I11.x–I13.x, I15.x
Chronic pulmonary disease		I27.8, I27.9, J40.x–J47.x, J60.x–J67.x, J68.4, J70.1, J70.3
Congestive heart failure		I09.9, I11.0, I13.0, I13.2, I25.5, I42.0, I42.5 I42.9, I43.x, I50.x, P29.0
Diabetes		E10.0, E10.1, E10.9, E11.0, E11.1, E11.9, E12.0, E12.1, E12.9, E13.0, E13.1, E13.9, E14.0, E14.1, E14.9, E10.2–E10.8, E11.2–E11.8, E12.2E12.8, E13.2–E13.8, E14.2–E14.8
Obesity		E66.x
Complication		
Intracranial hemorrhage		I60.x, I61.x, I62.x
Cardiac arrhytthmias		I44.1–I44.3, I45.6, I45.9, I47.x–I49.x, R00.0, R00.1, R00.8, T82.1, Z45.0, Z95.0
Cardiopulmonary resuscitation	8–771, 8–772, 8–779	
Embolism/thrombosis		I74.x
Myocardial infarction		I21., I22., I24
Pulmonary embolism		I26.x
Renal failure		I12.0, I13.1, N18.x, N19.x, N25.0, Z49.0Z49.2, Z94.0, Z99.2
Renal replacement therapy	8–853., 8–854., 8–855	
Stroke		I63, I64

Allocation of diagnosis related groups (DRG) and operation and procedure (OPS) codes as they are coded for reimbursement purposes in Germany.

Existing comorbidities (e.g. arterial hypertension, ICD I10.x, I11.x–I13.x, I15.x) and complications were defined by their respective ICD codes (Table 1).

### Statistical analysis

The data were descriptively analyzed. Categorical variables are expressed as absolute numbers and percentages. Due to the lack of median and quartiles in the data source, continuous variables were calculated using mean and standard deviation (SD). Due to the aggregated data, only group comparisons of categorical variables were possible. The OR were calculated by means of 2 × 2 frequency tables. For this purpose, the Pearson Chi square test with an assumed significance level of 0.05 was utilized and the OR was determined with the 95% confidence interval (CI). Excel 2019 (Microsoft Corp., Seattle, WA, USA) and Python with SciPy and the statsmodels (sm) package were used for the analyses.

### Ethics approval and consent to participate

Due to the institutional anonymization, no conclusions can be drawn about individual patients. According to §21KHEntgG the reimbursement data is free for scientific use. The Ethics Committee of the University Hospital Frankfurt waived the need for an Ethical Committee approval for this study (Chair: Prof. Dr. Harder, Ref: 2022-766). All data processing was performed in accordance with the Declaration of Helsinki.

### Consent for publication

As this are anonymised register data, no consensus of the patients can be collected.

## Results

A total of 561,379 patients were hospitalized with a confirmed SARS-CoV-2 infection in Germany from January 1st, 2020 to December 31th, 2021 and analyzed in this study.

### Patients’ characteristics

The proportion of female hospitalized patients was 47.65% (n = 267,516) overall. In total, 75.46% (n = 423,611) non-ICU and 24.54% (n = 137,750) ICU patients (Table 2). The biggest group was aged 80 years or older, being 30.07% (n = 168,779) overall. Divided in groups ICU and non-ICU and subgroups survivors and non-survivors: Patients aged 80 years or older had the highest conditional (relative) frequencies in non-ICU overall (32.17% (n = 136,270)), non-ICU-survivors (26.72% (n = 100,430)), non-ICU-non-survivors (75.10% (n = 35,840)), ICU overall (32.17% (n = 136,270)) and ICU-non- survivors (37.04% (n = 17,020)). The biggest age group in ICU-survivors was 65–74 years (22.63% (n = 20,772).

**Table 2:** Demographic data.

Demographic	Total in-patient		Admission to general ward		Admission to ICU	
	n	%	n	%	n	%
Total	561,379	100	423,611	75.46	137,750	24.54
Male	293,833	52.34	207,715	49.03	86,106	62.51
Female	267,516	47.65	215,874	50.96	51,636	37.49
Mortality	93,668	16.69	47,721	11.27	45,947	33.36
**Age groups**						
< 28 days	1105	0.20	901	0.21	204	0.15
28 days–1 year	3225	0.57	3050	0.72	175	0.13
1–2a	1704	0.30	1559	0.37	145	0.11
3–5a	1130	0.20	1021	0.24	109	0.08
6–9a	1352	0.24	1218	0.29	134	0.1
10–15a	2870	0.51	2613	0.62	257	0.19
16–17a	1781	0.32	1610	0.38	171	0.12
18–29a	22,400	3.99	19,887	4.69	2513	1.82
30–39a	34,052	6.07	28,928	6.83	5123	3.72
40–49a	43,916	7.82	34,096	8.05	9818	7.13
50–54a	33,878	6.03	24,920	5.88	8955	6.50
55–59a	42,169	7.51	29,751	7.02	12,417	9.01
60–64a	45,342	8.08	30,308	7.15	15,031	10.91
65–74a	97,435	17.36	64,845	15.31	32,588	23.66
75–79a	60,241	10.73	42,634	10.06	17,607	12.78
≥ 80a	168,779	30.07	136,270	32.17	32,503	23.60

Demographic data of inpatient in Germany from January 1st 2020 to December 31th 2021 with a positive SARS-CoV-2 PCR Test. Admission to general ward or admission to Intensive Care Unit (ICU).

### Length of in-hospital stay (LOS) comorbidities, complications and mortality rate

In all patients the LOS was 11.2 (SD = 12.2) days in 2020 and 11.7 (SD = 12.4) days in 2021. The most common comorbidity in all hospitalized patients was arterial hypertension (51.94% n = 291,577), followed by congestive heart failure (24.32% n = 136,505). In the non-ICU-group arterial hypertension was the most common comorbidity in survivors (47.82% (n = 179,738)) and non-survivors (61.79% (n = 29,489)), followed by congestive heart failure (17.25% (n = 179,738) in survivors and 61.79% (n = 29,489) in non-survivors, respectively. All other comorbidities and their frequencies in subgroups are displayed in Table 3.

**Table 3:** Comorbidities and complications.

			Overall						Non-ICU						ICU					
			Survivors		Non-survivors		p value	OR (95% CI)	Survivors		Non-survivors		p value	OR (95% CI)	Survivors		Non-survivors		p value	OR (95% CI)
Total	561,379		467,693		93,668				375,888		47,721				91,799		45,947			
	n	%	n	%	n	%			n	%	n	%			n	%	n	%		
**Comorbitiy**																				
Arterial hypertension	291,577	51.94	233,782	49.99	57,795	61.7	< 0.001	1.61 (1.58–1.64)	179,738	47.82	29,489	61.79	< 0.001	1.76 (1.73–1.80)	54,012	58.84	28,295	61.58	< 0.001	1.12 (1.10–1.15)
Chronic pulmonary disease	59,627	10.62	46,728	9.99	12,899	13.77	< 0.001	1.44 (1.41–1.47)	34,728	9.24	5327	11.16	< 0.001	1.23 (1.20–1.27)	11,975	13.04	7548	16.43	< 0.001	1.31 (1.27–1.35)
Congestive heart failure	136,505	24.32	91,366	19.54	45,139	48.19	< 0.001	3.81 (3.78–3.89)	64,853	17.25	22,360	46.86	< 0.001	4.23 (4.15–4.31)	26,496	28.86	22,772	49.56	< 0.001	2.42 (2.37–2.48)
Diabetes	40,830	7.27	29,807	6.37	11,023	11.77	< 0.001	1.96 (1.92–2.01)	21,978	5.85	5330	11.17	< 0.001	2.03 (1.96–2.09)	7751	8.44	5631	12.26	< 0.001	1.51 (1.46–1.57)
Obesity (total)	36,580	6.52	30,501	6.52	6079	6.49	0.726	1.00 (0.97–1.02)	19,330	5.14	1387	2.91	< 0.001	0.55 (0.52–0.58)	11,142	12.14	4686	10.2	< 0.001	0.81 (0.79–0.85)
WHO grade I	13,751	2.45	11,736	2.51	2015	2.15	< 0.001	0.83 (0.79–0.87)	8112	2.16	589	1.23	< 0.001	0.55 (0.50–0.60)	3619	3.94	1426	3.1	< 0.001	0.77 (0.72–0.82)
WHO grade II	8585	1.53	7221	1.54	1364	1.46	0.047	0.92 (0.86–0.97)	4530	1.21	298	0.62	< 0.001	0.50 (0.44–0.56)	2685	2.92	1063	2.31	< 0.001	0.78 (0.72–0.83)
WHO grade III	10,239	1.82	8309	1.78	1930	2.06	< 0.001	1.13 (1.08–1.19)	4689	1.25	309	0.65	< 0.001	0.50 (0.44–0.56)	3613	3.94	1621	3.53	< 0.001	0.88 (0.83–0.94)
**Complication**																				
Intracranial hemorrhage	2958	0.53	1434	0.31	1524	1.63	< 0.001	5.38 (5.00–5.78)	363	0.1	127	0.27	< 0.001	2.76 (2.25–3.38)	1042	1.14	1365	2.97	< 0.001	2.67 (2.46–2.89)
Cardiac arrhytthmias	160,881	28.66	112,247	24	48,634	51.92	< 0.001	3.42 (3.37–3.47)	80,036	21.29	21,946	45.99	< 0.001	3.14 (3.09–3.21)	32,203	35.08	26,667	58.04	< 0.001	2.56 (2.50–2.62)
Cardiopulmonary resuscitation	9728	1.73	2008	0.43	7720	8.24	< 0.001	20.83 (19.82- 21.89)	112	0.03	1184	2.48	< 0.001	85.36 (70.31–103.64)	1896	2.07	6536	14.23	< 0.001	7.86 (7.46–8.29)
Embolism/ thrombosis	2453	0.44	1525	0.33	928	0.99	< 0.001	3.06 (2.82–3.32)	529	0.14	184	0.39	< 0.001	2.75 (2.32 -3.25)	993	1.08	740	1.61	< 0.001	1.50 (1.36–1.65)
Myocardial infarction	6726	1.20	3987	0.85	2739	2.92	< 0.001	3.50 (3.34–3.68)	1706	0.45	913	1.91	< 0.001	4.28 (3.95–4.64)	2266	2.47	1814	3.95	< 0.001	1.62 (1.53–1.73)
Pulmonary embolism	12,730	2.27	8974	1.92	3756	4.01	< 0.001	2.14 (2.05–2.22)	4454	1.18	757	1.59	< 0.001	1.34 (1.24–1.45)	4520	4.92	2999	6.53	< 0.001	1.35 (1.28–1.41)
Renal failure	100,926	17.98	71,277	15.24	29,649	31.65	< 0.001	2.58 (2.54–2.62)	56,744	15.1	16,920	35.46	< 0.001	3.09 (3.03–3.15)	14,527	15.82	12,721	27.69	< 0.001	2.03 (1.98–2.09)
Renal replacement therapy	31,847	5.67	14,346	3.07	17,501	18.68	< 0.001	7.26 (7.09–7.43)	4398	1.17	884	1.85	< 0.001	1.59 (1.48–1.72)	9888	10.77	16,557	36.03	< 0.001	4.67 (4.54–4.80)
Stroke	5004	0.89	3286	0.7	1718	1.83	< 0.001	2.64 (2.49–2.80)	1036	0.28	412	0.86	< 0.001	3.15 (2.81–3.53)	2250	2.45	1301	2.83	< 0.001	1.16 (1.08–1.24)

Comorbidities and complications among SARS-CoV-2 positive patients in Germany from Jan 2020 to end of December 2021. The percentages refer column wise to the corresponding group.

*OR* odds ratio, *CI* confidence interval.

The most common complication was cardiac arrhythmia overall, coded in 28.66% (n = 160,881) of the patients overall and in every subgroup, as well. Especially in non-survivor group in ICU patients 58.04% (n = 26,667) and non-ICU patients 45.99% (n = 21,946), respectively. In each non-survivor group the rates were significantly higher, compared to the survivor group (Table 3).

Overall, 17.98% (n = 100,926) patients developed renal failure. The highest conditional (relative) frequency of patients needing dialysis was seen in non-survivors non-ICU-patients being 35.46% (n = 16,920). Cardiopulmonary resuscitation (CPR) was performed in 1.73% (n = 9728) of all COVID-19 inpatients. In non-survivor ICU-patients 14.23% (n = 6536) were treated with CPR. Pulmonary embolism occurred in 2.27% (n = 12,730) of all patients.

Our data shows an overall mortality rate of 16.69% (n = 93,668), 11.27% (n = 47,721) in non-ICU patients, and 33.36% (n = 45,947) in ICU-patients, respectively.

### Comparison survivors vs. non-survivors

The highest OR for comorbidity was seen in congestive heart failure (Overall: OR: 3.81 (3.78–3.89) non-ICU: OR: 4.23 (4.15–4.31) ICU: OR: 2.42 (2.37–2.48)). Obesity WHO grade I and II were the only comorbidities in which an OR < 1 was seen. In particular in obesity WHO grade I (Overall: OR: 0.83 (0.79–0.87), non-ICU: OR: 0.55 (0.50–0.60), ICU: OR: 0.77 (0.72–0.82)) (Table 3).

There is a significant difference between the groups of non-survivor/survivor within all complications (p < 0.001). The highest OR for the non-survivor groups were seen in CPR (Overall: OR: 20.83 (19.82–21.89) non-ICU: OR: 85.36 (70.31–103.64) ICU: OR: 7.86 (7.46–8.29)). Followed by renal replacement therapy in patients overall (OR: 7.26 (7.09–7.43)), renal failure in non-ICU patients (OR: 3.09 (3.03–3.15)) and embolism or thrombosis in non-ICU patients (OR: 2.75 (2.32–3.25)). (Table 3).

### Analysis in the context of time course

**Figure 1:**
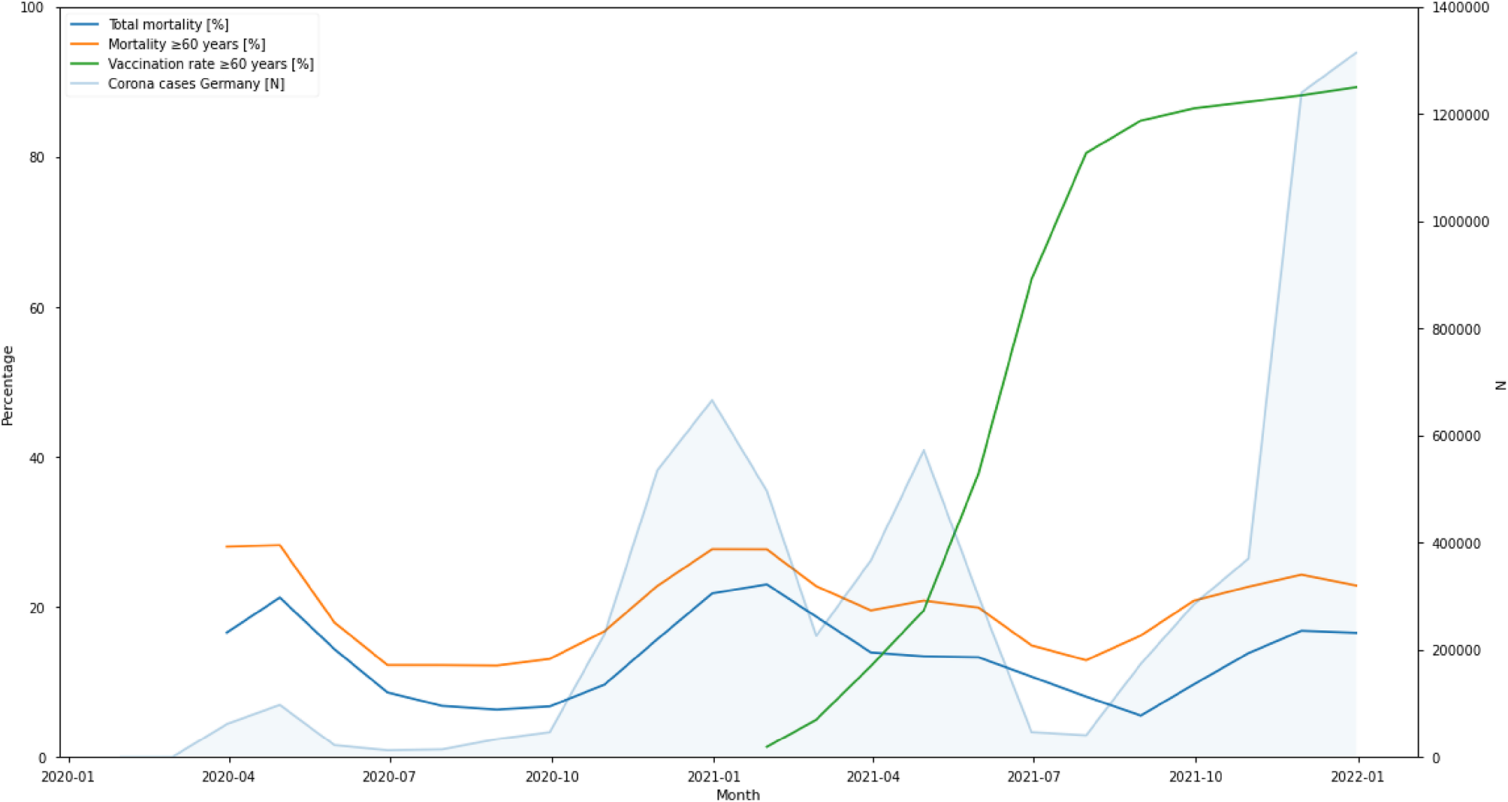
Mortality, vaccination and corona cases over time in Germany. Mortality of in hospital patients compared to COVID-19 vaccination and number of COVID-19 cases in Germany over time. Corona cases in Germany [n]. Fraction of mortality for $\ge$ 60 years [%]. Vaccinated percentage of age group $\ge$ 60 years [%] and total mortality [%] over time.

The observed in-hospital mortality-rates fluctuated during the analyzed and showed three peaks: April 2020 and January 2021 being 21.23% (n = 4539) and 22.99% (n = 15,724) and in November, December 2021 (16.82%, n = 7173 and 16.54%, n = 9416) (Fig. 1, Table 4).

**Table 4:** Mortality.

	_Mortality_	_Mar 20_	_Apr 20_	_May 20_	_Jun 20_	_Jul 20_	_Aug 20_	_Sep 20_	_Oct 20_	_Nov 20_	_Dec 20_	_Jan 21_	_Feb 21_	_Mar 21_	_Apr 21_	_May 21_	_Jun 21_	_Jul 21_	_Aug 21_	_Sep 21_	_Oct 21_	_Nov 21_	_Dec 21_
		_16.58_	_21.23_	_14.35_	_8.61_	_6.82_	_6.32_	_6.75_	_9.65_	_15.70_	_21.78_	_22.99_	_18.69_	_13.93_	_13.40_	_13.29_	_10.71_	_8.05_	_5.53_	_9.68_	_13.84_	_16.82_	_16.54_
_Age groups_	_<28 days_	_0.00_	_0.00_	_0.00_	_0.00_	_0.00_	_0.00_	_0.00_	_0.00_	_0.00_	_0.00_	_0.00_	_0.00_	_0.00_	_0.00_	_0.00_	_0.00_	_0.00_	_0.00_	_0.00_	_0.00_	_0.00_	_0.00_
	_28 d–1 year_	_0.00_	_0.00_	_0.00_	_0.00_	_0.00_	_0.00_	_0.00_	_0.00_	_0.00_	_0.00_	_0.61_	_0.00_	_0.00_	_0.00_	_0.61_	_0.00_	_2.70_	_0.00_	_0.00_	_0.00_	_0.00_	_0.21_
	_1–2_	_0.00_	_0.00_	_0.00_	_0.00_	_8.33_	_0.00_	_0.00_	_0.00_	_0.00_	_0.00_	_0.00_	_0.00_	_0.00_	_0.56_	_1.83_	_0.00_	_0.00_	_0.00_	_0.00_	_0.00_	_0.50_	_0.94_
	_3–5_	_0.00_	_6.25_	_0.00_	_0.00_	_0.00_	_0.00_	_0.00_	_0.00_	_0.00_	_0.00_	_0.00_	_0.00_	_1.41_	_0.00_	_2.78_	_0.00_	_0.00_	_0.00_	_0.00_	_1.47_	_0.76_	_1.17_
	_6–9_	_0.00_	_0.00_	_0.00_	_0.00_	_0.00_	_0.00_	_0.00_	_0.00_	_1.27_	_1.85_	_0.00_	_0.00_	_0.00_	_0.00_	_0.00_	_0.00_	_0.00_	_0.00_	_0.00_	_1.59_	_0.68_	_0.00_
	_10–15_	_0.00_	_0.00_	_0.00_	_0.00_	_0.00_	_0.00_	_0.00_	_1.27_	_0.44_	_0.00_	_0.00_	_0.00_	_0.00_	_0.00_	_1.14_	_0.00_	_0.00_	_0.00_	_0.56_	_1.29_	_0.00_	_0.44_
	_16–17_	_0.00_	_3.70_	_0.00_	_0.00_	_0.00_	_0.00_	_0.00_	_0.00_	_0.00_	_0.00_	_0.00_	_0.00_	_0.00_	_0.00_	_0.00_	_0.00_	_0.00_	_0.00_	_0.00_	_0.00_	_0.58_	_1.00_
	_18–29_	_0.79_	_0.37_	_1.20_	_0.00_	_0.00_	_1.44_	_0.00_	_0.23_	_0.48_	_0.26_	_0.55_	_0.55_	_0.59_	_0.42_	_0.91_	_0.23_	_0.29_	_0.16_	_0.92_	_0.57_	_0.82_	_0.82_
	_30–39_	_0.24_	_0.95_	_1.79_	_0.93_	_0.00_	_0.44_	_0.75_	_0.56_	_0.48_	_1.00_	_1.10_	_1.49_	_1.46_	_0.97_	_1.56_	_2.14_	_1.40_	_0.55_	_1.42_	_2.01_	_1.28_	_1.63_
	_40–49_	_1.05_	_2.42_	_3.87_	_1.70_	_2.39_	_0.58_	_0.52_	_1.06_	_1.56_	_2.27_	_3.11_	_2.97_	_2.10_	_2.24_	_3.60_	_4.11_	_3.43_	_1.52_	_2.80_	_3.36_	_3.14_	_3.90_
	_50–54_	_3.09_	_4.66_	_4.74_	_3.55_	_2.14_	_2.40_	_2.02_	_1.34_	_2.70_	_4.14_	_6.03_	_5.96_	_3.68_	_4.33_	_5.22_	_6.72_	_7.98_	_1.60_	_4.63_	_4.72_	_5.80_	_7.43_
	_55–59_	_4.28_	_6.87_	_7.79_	_5.11_	_3.41_	_3.46_	_2.75_	_2.36_	_4.18_	_6.70_	_8.91_	_7.43_	_5.58_	_6.23_	_8.27_	_9.35_	_9.17_	_4.46_	_5.72_	_8.31_	_8.04_	_10.28_
	_60–64_	_7.25_	_11.48_	_8.18_	_8.84_	_8.75_	_6.13_	_4.15_	_3.70_	_7.12_	_11.33_	_12.02_	_10.93_	_8.72_	_9.30_	_10.11_	_11.42_	_10.39_	_7.41_	_9.40_	_11.20_	_11.69_	_12.98_
	_65–74_	_12.97_	_18.97_	_15.01_	_10.93_	_8.25_	_7.76_	_6.77_	_8.56_	_12.28_	_17.75_	_20.14_	_16.75_	_14.01_	_16.52_	_17.07_	_14.53_	_13.41_	_12.49_	_15.92_	_17.09_	_17.13_	_19.29_
	_75–79_	_26.04_	_27.86_	_18.27_	_12.04_	_15.57_	_14.38_	_18.01_	_14.39_	_20.95_	_25.01_	_24.56_	_22.23_	_19.57_	_22.63_	_22.78_	_15.41_	_14.61_	_17.56_	_23.85_	_20.00_	_22.71_	_21.24_
	_≥ 80_	_49.69_	_38.33_	_21.43_	_14.30_	_15.14_	_19.21_	_20.93_	_28.50_	_33.22_	_35.72_	_34.20_	_27.78_	_26.40_	_29.72_	_27.59_	_17.08_	_13.30_	_24.15_	_28.26_	_30.38_	_31.91_	_28.52_
_Sex_	_Male_	_18.48_	_23.41_	_16.59_	_9.20_	_7.99_	_7.32_	_7.80_	_10.64_	_17.27_	_24.41_	_25.85_	_21.26_	_15.46_	_15.06_	_15.04_	_12.38_	_8.70_	_6.17_	_10.64_	_15.38_	_18.77_	_18.84_
	_Female_	_13.92_	_18.59_	_11.76_	_7.86_	_5.26_	_5.12_	_5.54_	_8.53_	_13.98_	_19.09_	_20.06_	_15.96_	_12.22_	_11.47_	_11.16_	_8.65_	_7.27_	_4.86_	_8.62_	_12.17_	_14.73_	_13.98_

Mortality [%] over time by age subgroups from January 2020 to end of December 2021.

Within the whole-time patients aged 60 years or older had the highest mortality in all hospitalized patients. Analyzed by the time trend of the data divided into months from January 2021 until the end of December 2021, in every month the highest mortality was observed in the age group 80–85 years, with particularly high mortality in months when hospitalization rates were the highest (Fig. 1, Table 4).

## Discussion

This retrospective study includes a cohort of 561,379 hospitalized patients from January 1st, 2020 to December 31th, 2021 with a positive SARS-CoV-2 PCR test. Our main findings are a mortality rate of 16.69% overall. Between months of the ongoing pandemic, the mortality fluctuates. Especially in the “third wave”, a noticeable decrease in the proportion of elderly patients was seen.

The COVID-19 pandemic in Germany in the analyzed period can be described in four waves: the first one from March until May 2020^12^, the second one from October 2020 until January 2021^12^ and a third wave from March until April 2021^13^. In the end of 2021 Germany went through the fourth wave. The cause of the undulating course of infection numbers is multifactorial, main reasons are higher temperatures in spring/summer^14^, social distancing and shutdown measures by the government^13^.

### Mortality rate

The mortality rate of 16.69% overall is lower than previously described mortality-rates. Richardson et al. described 5700 hospitalized patients diagnosed with COVID-19 in New York from March until April 2020 and found a mortality rate of 21%, overall but a particularly lower rate of ICU-patients (12.2%)^15^. Karagiannidis et al. described over 10,000 patients suffering from COVID-19 in Germany from February until April 2020 in Germany and found a mortality rate of 22% overall^16^. This might be explained by the fact that previous studies describe a shorter time period. In December of 2020 the vaccine against COVID-19 was licensed in Germany^17^. Over time, more and more treatment options are being explored, and guidelines for the treatment of COVID-19 infection continue to evolve on a regular basis.

Due to prioritization rules the vaccines were only available for patients over 75 years of age, employees in high-risk facilities and patients with defined comorbidities were vaccicinated^17^. The prioritization was lifted in May 2021. The first “vaccination effect” may occur in January 2021, which could explain the decreasing proportion of patients aged 60 years or older from there on.

In contrast to that we found higher mortality rates in ICU patients, being 33.36%. A possible explanation might the higher capacity of ICU-beds in germany^16^ and therefore the possibility to admit patients with more comorbidities or at higher age to the ICU, while in some countries ICUs reached their capacity limit^3^.

Our data shows a distinct mortality between minors (< 18 years) and old adults (> 60 years).

Compared to the German population (17%, in 2020) and the share of SARS-CoV-2 infected persons (29%), the group of children (< 18 years) is significantly underrepresented among all hospitalised patients (2.35%, Table 2)^18,19^. Furthermore, 9% (< 18 years) against 26% (> 60 years) conditional on the respective group were admitted to ICU. There a various explanation for this finding. Children have less comorbidities such as obesity, diabetes, cancer and other chronic diseases. Children and adults show a different immune response to a viral infection, protecting children from severe COVID-19^20^. Our findings can also be explained with a difference in the expression of ACE2-receptor, the primary receptor of SARS-CoV-2^21^. Children express lower levels of ACE2^21^ and therefore have fewer symptoms and a better prognosis^22^.

### Comorbidities

Arterial hypertension was the most common comorbidity in our study (51.94% (n = 291,577) overall), this result is in line with other studies describing hypertension as the most common comorbidity with rates of 42%^23^ up to 56%^16^.

Obesity is known to be a risk factor for COVID-19^24^ and is associated with higher mortality^11^. The incidence of 6.52% for obesity is lower than rates described in the UK (10.2%)^11^ or France (47.6%)^25^. Surprisingly our data showed a reduced odd for death in patients with low grade obesity, especially WHO grade I (non-ICU: 0.55 (0.50–0.60) and ICU: 0.77 (0.72–0.82), respectively). Dana et al. found a lower risk of death for critically ill COVID-19 patients among patients with moderate obesity^26^. This finding is not in line with other studies^27^. However, the Centers for Disease Control and Prevention (CDC) states that patients at the “threshold” between healthy weight and overweight are at lower risk for hospitalization, ICU admission, and death while the risk for mechanically ventilation raised continuously with rising body mass index (BMI)^28^. Our findings support the statement of the CDC. Patients with a low grade overweight (WHO grade I) have a higher chance of survival (With increasing obesity, the effect is lost and the overweight becomes a risk factor for the patient (WHO grade III, OR: 1.13 (1.08–1.19)). However, overweight and moderate obesity are known to have a protective effect on hospitalized non-COVID-19 ICU-Patients^29^. This effect is not yet fully understood and often controversially referred to as “obesity paradox”^30^. With a lower rate of high grade obese patients admitted to the hospital, more patients are at the “threshold” described by the CDC. Foo et al. described for every 1% increase in obesity prevalence, the COVID-19 mortality rate was increased by 8.3%^31^. This might explain our finding.

We could find a significant difference in the frequency of patients suffering from congestive heart failure between survivors and non-survivors, with a bigger frequency of heart failure in non-survivors. One possible explanation is that patients suffering from heart failure have a higher overall frailty and are at higher risk for acute cardiac injury. Several studies investigated cardiovascular manifestations and mechanisms in patients suffering from COVID-19 and showed a high prevalence of cardiovascular comorbidities^32^ and a higher risk for mortality in patients suffering from cardiovascular comorbidities or cardiac injury^32,33^. Jabri et al. described a significant increase in stress cardiomyopathy during the pandemic^34^, furthermore a frequent occurrence of cor pulmonale in COVID-19 patients has been described^35^. All this factors explain the high proportion of cardiac comorbidities in hospitalized patients and the increase of those diagnoses in the non-survivor groups.

### Complications

We found arrhythmias to be the most common complication in our study. We showed a rate of 42.74% (n = 58,870) for ICU patients. This result is comparable to a study by Duo et al. who described a rate of 44.4% among ICU patients in a study investigating 138 patients overall^32^.

Electrocardiographic abnormalities are often described in patients suffering from COVID-19^36,37^.

One underlying reason may be the mismatch between oxygen supply and oxygen demand resulting in cardiac injury. Therefore, the high OR for death is not surprising.

Thrombotic events are often seen in COVID-19 patients. Those events are associated with a more severe disease and increased mortality^38,39^. The molecular explanation range from COVID-19-associated coagulopathy to genetic predisposition and is still subject to research^40,41^. Our findings are in line with literature, we showed a pulmonary embolism rate of 2.27% (n = 12,730) for all hospitalized COVID-19 patient. Patients suffering from pulmonary embolism have a higher chance of dying (OR: 2.14 (2.05–2.22)). OR on ICU is lower (OR: 1.35 (1.28–1.41). One explanation for this could be that more radiological examinations are performed on ICU and therefore associated with a higher number of incidental findings without clinical relevance^42^. A second explanation is that patients on ICU are more likely to be sufficiently anticoagulated, as recommended in guidelines^43,44^. Unfortunately, medication application such as anticoagulation is not provided in the reimbursement data.

Renal failure is a common complication in patients suffering from COVID-19^15,45^. The observed rate of patients with acute kidney failure (17.98% n = 100,926) is in line with other studies, describing rates between 20 and 40%^15,45^. The higher proportion of patients with renal replacement therapy (RRT) in the ICU-group might be explained by the fact that needing dialysis is a common reason to be admitted to the ICU. Patients who had been diagnosed with acute kidney failure and a following admission to the ICU are displayed in the ICU-group only. In our study 5.67% (n = 31,847) patients needed RRT, this result seems to be higher than in other studies, for example Richardson et al. described a rate of 3.2%.

COVID-19 patients are more likely to suffer from in-hospital-cadiac-arrest (IHCA)^46^. In our study 1.73% (n = 9728) of all patients received CPR. The OR of dying was remarkably higher on the normal ward compared to the ICU. This is in accordance to the literature. Acharya et al. described a IHCA for non-ICU patients in 2.2% (in our study: 2.48%) and for ICU patients in 15.4% (in our study 14.2%)^47^. A possible cause for the high difference between non-ICU and ICU patients might be the longer timespan between the circulatory arrest and the actual start of CPR due to the lack of monitoring^48^.

This study is the first in Germany to describe the course of the pandemic from the beginning to the end of December 2021, covering 561 379 hospitalized COVID-19 Patients.

Further studies should be conducted to identify risk factors for an adverse course of the disease. On one hand, this could help to identify patients with a special risk profile at an early stage (for example Simonnet et al. investigated obesity as a risk factor^25^) or, on the other hand, serve as criteria in possible triaging.

## Conclusion

The overall mortality rate of 16.69% in COVID-19 patients in Germany is lower than previously shown in other studies. 24.54% of all patients had to be treated on the ICU with a mortality rate of 33.36%, which was high in comparison to the literature. Congestive heart failure was associated with a significantly higher risk of death. In non-ICU patients suffering from congestive heart failure the OR was especially high, being 4.23 (4.15–4.31). The most common comorbidity in all COVID-19 patients was arterial hypertension. The most common complication were arrhythmias, which were diagnosed significantly more often in non-survivors (p < 0.001). In COVID-19 patients CPR is associated with a high chance of death, especially on normal wards (OR: 85.36 (70.31–103.64)). With an OR: 0.83 (0.79–0.87) WHO grade I obesity might have a protective effect of the patient’s survival.

Pre-existing cardiac conditions appear to carry a particularly high risk in patients suffering from COVID-19.

Due to the limited data, no further research could be done, so it is extremely important to make this data available to the scientific community in order to gain a wider insight and better understand possible causal relationships.

### Limitations

Due to the provision of data by the InEK during the year, only highly aggregated data are available and no further detailed queries are possible. Patients’ age is only available in the provided subgroups, so no further analysis like mean or median age can be made. As the data (e.g. comorbidities) were anonymized and cannot be tracked back to a single patient further analysis such as linear regression are not possible. Laboratory findings or medication are not coded for reimbursement and are therefore not available for analysis.

## Data Availability

The German Institute for Hospital Remuneration System (InEK) supports hospitals and health insurance funds as well as their associations in the introduction and continuous further development of the German-DRG system in accordance with the Krankenhausfinanzierungsgesetz (KHG; Hospital Financing Act). Since 2020, access to these data has been possible during the year. For this observational study, we used publicly accessible performance data provided by InEK. Since the register data were anonymized, the Ethics Committee of the University Hospital Frankfurt waived the need for an Ethical approval (Chair: Prof. Dr. Harder, Ref: 2022-766).

## Abbreviations

CDC: Centers for Disease Control and Prevention
CI: Confidence interval
COVID-19: Coronavirus disease 2019
CPR: Cardiopulmonary resuscitation
DRG: Diagnosis-related group
ICD: International classification of diseases
ICU: Intensive care unit
IHCA: In-hospital-cardiac-arrest
InEK: German Institute for Hospital Remuneration System
KHG: Krankenhausfinanzierungsgesetz (Hospital Financing Act)
LOS: Length of in-hospital stay
Non-ICU: Non-intensive care unit (= general ward)
OPS: Operation and procedure code
RT-PCR: Real-time-polymerase chain reaction
